# Bone Lid Technique Using a Piezoelectric Device for the Treatment of a Mandibular Bony Lesion

**DOI:** 10.1155/2017/9315070

**Published:** 2017-12-07

**Authors:** Ronald Younes, Ibrahim Nasseh, Pierre Lahoud, Elie Wassef, Maroun Dagher

**Affiliations:** ^1^Department of Oral Surgery, School of Dentistry, Saint Joseph University, Beirut, Lebanon; ^2^Department of Radiology, Lebanese University, Beirut, Lebanon; ^3^Department of Periodontology, School of Dentistry, Saint Joseph University, Beirut, Lebanon

## Abstract

Different techniques for the enucleation of jaw cyst lesion in the oral and maxillofacial regions have been proposed, including the bone lid technique. The purpose of this case report is to describe the case of a cystic lesion, approached with the bone lid technique performed using a piezoelectric device, with an 8-month clinical and radiographic follow-up. A 14-year-old male patient was treated for a suspicious lesion detected on a panoramic radiograph. The concerned area was surgically accessed, and a radiographically predetermined bony window was drawn, and the beveled bony lid was removed. The underlying lesion was enucleated and sent for pathology as a routine procedure, and the removed bony lid was repositioned in situ and secured with a collagen tape. Healing was uneventful with limited swelling and reduced pain. A complete radiographic bone healing at the previously diseased site was confirmed with an 8-month cone beam computed tomography (CBCT) scan with no buccal bone resorption nor ridge collapse. The bone lid technique with a piezoelectric device was noninvasive and atraumatic in this case. Further studies are needed and could lead to the adaptation of this approach as a possible standard of care.

## 1. Introduction

Jaw lesions are common in the oral and maxillofacial regions [1]. Their enucleation and the primary closure of the defects, the so-called “cystectomy” has evolved as the treatment of choice or standard of care to the present day [2]. The result of such treatment leads to the formation of a bone cavity that is left to heal. The starting point of healing in such cases is the formation of a blood clot that will lead to a granulation tissue which will be replaced by the bone via the process known as osteogenesis. In general, osteogenesis starts from the periphery of the defect towards the center. The endosteum or the marrow spaces offer osteoblasts that are going to differentiate and deposit bone.

When the remaining osseous defect is small in size, the newly formed blood clot is substituted within few weeks by an immature bone, while as in large bone defects, this process may take many months. Sometimes, after a big cyst removal is achieved, an incomplete bone regeneration occurs, and the cavities are filled with scar tissues instead of a complete newly formed bone. And in some extreme cases, an incomplete bone healing may remain for years [3]. This is usually the result of an originally large extended bone lesion, and the need to gain access and visibility to this big lesion that entails a large osteotomy leaves a big bone defect with an absent buccal bone that could have eventually been a source for future osteoblasts.

Different alternative techniques or approaches for the treatment of big cystic lesions have been proposed, including the bone lid technique. Originally described for apicoectomies, the bone lid technique consisted of cutting a window into bone, creating a lid, removing it, accessing the desired area, and replacing the lid in situ, fixated or not, depending on the need [4]. The advantage of this technique, particularly in big bony defects, is that it offers a secluded space, whereas osteogenic cell population originating from the internal walls of the defect can easily repopulate the osseous wound, while forbidding the nonosteogenic cell population, mainly the epithelium, fibroblast, and other connective tissue cells to invade the cavity. This follows the biological concepts and fundamental principles of guided bone regeneration [5, 6]. The aforementioned needed bone lid was usually created using burs, or microsaws [7]. Lately, piezoelectric devices have been introduced with multiple applications, including bone lid creation [8–10].

The purpose of this technical note is to describe the case of a bony lesion, treated using the bone lid technique with a piezoelectric device, with a 12-month radiographic follow-up.

## 2. Case Report

**Figure 1:**
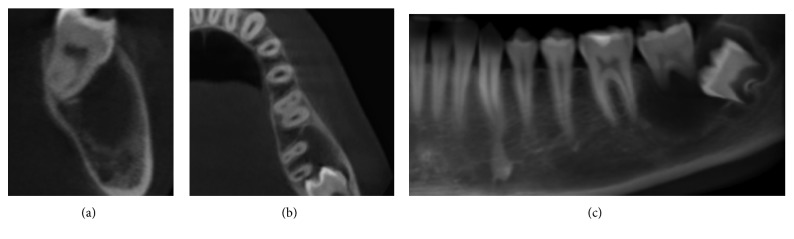
Preoperative radiographic examination: CBCT panoramic reconstruction, showing low-density image extending from the distal root of 36 (a), englobing the radicular part of 37 and the crown of 38 at baseline; axial cut showing extension of the lesion in buccolingual view, with thinning of the buccal cortical without interruption and displacement of 37 in the lingual direction; cross-sectional view at the level of 37 (b), showing clearly the displacement in lingual direction (c).

A 14-year-old male patient was referred for suspicion of a mandibular cystic lesion in the site of tooth number 37 where the lesion was incidentally discovered. Clinically, the patient presented no signs of infection, no pain, and no history of swelling. Radiographically, it extended from the distal part of 36, englobing the root of 37 and the crown of 38, invading the body of the mandible within the limits of the buccal and lingual cortices without perforation, and extending apically close to the inferior alveolar canal (Figure 1).

**Figure 2:**
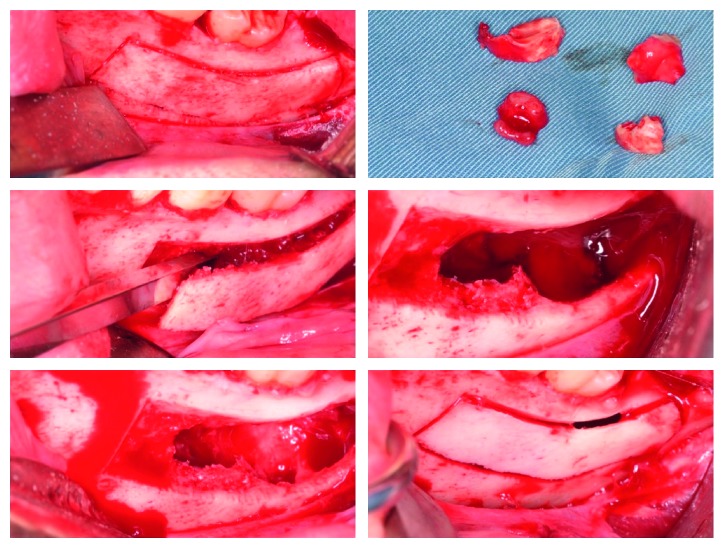
Bone lid was prepared using the piezoelectric device, bone lid was freed using an angulated bone chisel, lesion was removed, and bone lid was replaced in situ.

Following administration of local anesthetic, an intrasulcular incision was made on teeth number 36, 37, and in the site of 38, extending distally on the anterior border of the ramus with continuous contact with the bone. Full-thickness flap was reflected to gain access to the underlying bone. A bony window was drawn extending at least 5 mm more than the originally radiographically predetermined size of the lesion using the OT7 tip mounted on the piezoelectric device. This extension of the bone window will secure a latter repositioning of the lid on a healthy stable bone. The used tip was directed in a beveled orientation through the healthy external cortical plate down to the cancellous bone, as denoted by the reduced resistance to pressure. The beveled bony lid was freed with an angulated bone chisel using a gentle luxation with progressive movements in order to avoid any possible fracture (Figure 2). The bony lid was thus removed and placed in a sterile saline solution. The underlying lesion was visualized and enucleated using curettes and sent for pathology as a routine procedure. Careful complete removal of the lesion was achieved, ensuring a total absence of any soft tissue remnant inside the bone cavity that was rinsed repeatedly with intraoral Betadine® (povidone-iodine). After wisdom tooth extraction, the removed bony lid was repositioned in situ. Digital pressure was used trying to move the lid in all directions, thus ensuring its proper repositioning in its original place and optimizing its stability (Figure 2).

Nevertheless, it was secured with a collagen tape (CollaTape®) that acted as an extraplugging material. The flap was sutured in place in a tension-free manner using a 4.0 resorbable vicryl suturing material. Postoperative medication included Augmentin® 625 mg, BID for 7 days, ibuprofen 400 mg, TID for 3 days, and a chlorhexidine mouthwash (0.12%), TID for 2 weeks. Healing was uneventful as reported by the patient with limited swelling and reduced pain.

**Figure 3:**
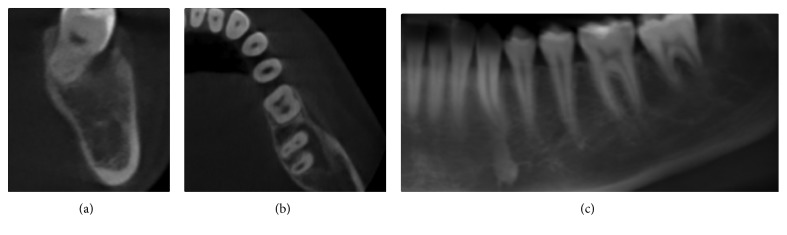
8-month radiographic examination: CBCT panoramic reconstruction, showing partial healing of the lesion and normal eruption of 37 (a); axial cut showing partial healing of the lesion and almost normal position of 37 (b); cross-sectional view at the level of 37 showing partial healing of the lesion (c).

A routine 8-month cone beam computed tomography (CBCT) was done. It revealed an almost complete radiographic bone healing at the previously diseased site (Figure 3).

Interestingly, no buccal bone resorption was noted nor any ridge collapse.

## 3. Discussion

We presented a case of a mandibular lesion that was treated using the bone lid technique with a piezoelectric device. The bone lid technique, per se, has been used in different clinical situations, including periapical surgeries and implant-related surgeries [11, 12]. Cho et al. [7] demonstrated that a bony window when repositioned in place after sinus grafting works as a barrier membrane with additional osteogenic effects. Thus, this autogenous bony membrane increases the external cortical healing, and consequently, a higher percentage of vital bone formation will occur. In our case, the repositioned bone lid technique clearly prevented a buccal bone depression while optimizing a structural restitution of the defect. This was confirmed by our 8-month clinical and radiographic findings (Figure 3).

The bone lid also helped create and maintain a secluded volume where a blood clot could form and ultimately lead to an almost complete bone healing. This seems to follow the basic principle of guided bone regeneration where a cell occlusive membrane is used in order to create and seal off a space favorable for the exclusive recruitment and proliferation of osteoprogenitor cells, while preventing the entry of nonosteogenic cells and ultimately leading to complete bone formation [13, 14]. Different membrane materials have been used, including resorbable and nonresorbable membranes. We used the bone lid as a rigid autogenous membrane.

Of the most common approaches to the bone lid technique were the rotary instruments and the microsaws, but nowadays, the piezoelectric device has proven to be a safe and efficient tool in different surgical situations [15, 16]. In the present case, we applied the bone lid technique using a piezoelectric device. Previous studies and reports demonstrated the advantage of using a piezoelectric device [8, 17–19], including a favorable osseous repair and remodeling when compared to carbide bur and diamond bur osteotomies [20]. In this case, the piezoelectric device allowed a selective cutting action of the mineralized tissue, while avoiding any impinging on the underlying soft tissue, including the cystic envelope.

In the same conservative spirit, tooth number 37 was not extracted at the time of surgery. The young age of the patient encouraged us to conserve this tooth, regardless of its original radiographic ill-positioning. Our results confirmed the appropriateness of this therapeutic decision.

The application of a bone lid technique using a piezoelectric device in a conservative approach to treat a cystic lesion seemed to have contributed to a proper bone formation into the defect while maintaining the outer jaw bony contour, with a short-term (8 months) successful outcome as herein presented. Further studies are needed and could lead to the adaptation of this approach as a possible standard of care.
